# Editorial: Linking the endocrine system with immunity

**DOI:** 10.3389/fendo.2025.1638965

**Published:** 2025-08-15

**Authors:** Dorota Latek, Yildiz Tutuncu

**Affiliations:** ^1^ University of Warsaw, Faculty of Chemistry, Warsaw, Poland; ^2^ Division of Medical Biology, School of Medicine, Koc University, Istanbul, Türkiye

**Keywords:** immune system, CD8+ T cells exhaustion, gestational diabetes mellitus, cre-lox system, glucocorticoid, collision tumor, endocrine system, lipopolysaccharide-induced inflammation

The immune and endocrine systems are considered two interconnected networks that influence each other to maintain biotic homeostasis. This Research Topic aims to present the dynamic interaction between innate and adaptive immunity and the endocrine system in various physiological and pathological conditions.

The immune system is a mechanism of protection that simultaneously maintains the organism’s homeostasis. Stress-induced activation of the hypothalamic-pituitary-adrenal axis (HPA) is a double-edged sword, activating or suppressing the immune system in specific tissues and cells and affecting innate and adaptive immune responses as described in detail by (Xu et al.). Corticosterone, a glucocorticoid (GR) that regulates the HPA axis, affects immunity through an overwhelming number of signaling pathways, from GR signaling and transcription regulation, through endoplasmic reticulum stress signaling, to toll-like receptor signaling.

It is known that diabetes elevates HPA axis activity, causing increased release of ACTH, which stimulates the adrenal gland to produce glucocorticoids. However, the molecular mechanisms underlying this steroidogenesis are still not fully understood. Magalhães et al. investigated the effects of toll-like receptor 4 (TLR4) pathway activation via lipopolysaccharide (LPS) derived from intestinal bacteria on adrenal steroidogenesis in diabetic mice. Experiments with a TLR4 antagonist (TAK-242) and a mouse model of alloxan-induced diabetes, first described in 1943 by Shaw Dunn and McLetchie as alloxan-induced damage to β-cells in rats (1), showed that corticosterone levels were significantly reduced. These findings suggest that TLR4 activation by bacterial LPS contributes to adrenal steroidogenesis in diabetes and may serve as a novel target for managing glucocorticoid-related complications.

The autoantigen-driven clonal amplification of T cells is recognized as the hallmark of T1D pathophysiology. Only 20 stem cell-like CD8+ T cells inducing type 1 diabetes (T1D) in healthy mice (2) and modulated self-reactive CD8+ T cells used to revert it are mentioned by Yang et al.. Stem cell-like progenitor CD8+ T cells in the pLN replenish the short-lived population of pathogenic T cells that directly destroy β-cells and cause T1D (2). Prevention and treatment of T1D by restoring or inducing immune tolerance to β-cells requires a precise description of the mechanisms underlying the state changes of CD8+ T cells. New anti-T1D therapies include enhancing the therapeutic CD8+ T cell exhaustion, CRISPR/Cas9-based gene editing in CD8+ T cells, CAR-T engineering of T cells, and T cell differentiation by single-cell RNA sequencing or single-cell transcriptome analysis combined with T cell receptor sequencing. Yang et al. emphasized that the best therapy for T1D is to regulate the autoimmune T cell response while maintaining a proper immune system response to foreign antigen invasion and avoiding systemic immunosuppression (3, 4).

Any risk associated with gestation negatively affects the steadily declining birth rate in Europe (3.67 million births in 2023 with almost half of children born to first-time mothers) (5). Gestational diabetes mellitus poses a significant but differential risk in certain regions and Ray et al. discussed its genetic, gene-environment, and inflammatory causes, describing genetic variants of SLC30A8, CDKAL1, TCF7L2, IRS1, and GCK along with the inflammatory pathways NF-κB, TNF-α, and IL-6.

A unique, lipopolysaccharide-induced, chronic inflammation mouse model, different from others (6), with implications for understanding the impact of endotoxemia on reproduction, was demonstrated by (Garcia et al.). Essential for reproduction, luteinizing (LH) and follicle-stimulating hormone (FSH) levels were elevated upon LPS-mediated stimulation of the TLR4 signaling cascade, leading to paracrine activation of TGF-β pathways. Furthermore, RNA-sequencing revealed LPS-induced changes in the pituitary indicating local paracrine mechanisms for FSH upregulation with TGF-β2 as an important factor. However, transcripts for LH and FSH decreased or remained unchanged suggesting that the secretion of these gonadotropins is independent of transcription but is rather induced by the pituitary microenvironment signaling. Garcia et al. discussed their results obtained with chronic, 6-week, low-dose LPS stimulation contrasting with previous studies showing suppression of the hypothalamic-pituitary-gonadal (HPG) axis in mice treated with high-dose and acute LPS.

Unexpected phenotypes in mouse models leading to contradictory or unconvincing conclusions were discussed in another article. Rahman et al. discussed complications that can be introduced with the Cre-Lox system (7, 8) in knockout mouse models. They compared two KO mouse models, Lyz2Cre and Nod1 floxed, and observed that myeloid Cre expression alone was enough to induce an anti-inflammatory phenotype protecting against palmitate-induced glucose intolerance. Notably, Lyz2Cre reduced absolute insulin secretion, increased insulin clearance, and increased insulin sensitivity in the absence of palmitate infusion. Furthermore, Lyz2Cre expression impaired bone marrow-derived macrophage function. Rahman et al. suggest that these observed effects could be due to Cre toxicity, haploinsufficiency of the Lys2 locus, or the effect of non-specific Cre expression in distinct locations and emphasize the role of appropriate controls while using the Lyz2Cre model.

Autoimmune diseases often occur with comorbidities of either autoimmune origins such as Sjögren’s syndrome or non-autoimmune pathophysiology such as cardiovascular or kidney diseases. Cardiovascular and respiratory diseases are the most common cause of death in patients with rheumatoid arthritis (9, 10). Hyperuricemia is known as the pathological basis of gout, an inflammatory joint disease, and can lead to the occurrence and progression of comorbidities such as hypertension, diabetes, chronic kidney disease, cardiovascular diseases, etc. Zhang et al. reported that patients with increased inflammation response index (SIRI) and systemic immune-inflammation index (SII), integrating three independent white blood cell subsets and platelets, had also an elevated risk of hyperuricemia with increased BMI as a mediating effect. Another link between different types of autoimmune diseases was discussed by Wu et al. using hypothyroidism as an example. Hypothyroidism is associated with a reduced risk of inflammatory bowel disease, specifically Crohn’s disease.

The most prevalent autoimmune diseases affecting endocrine glands are hypothyroidism (Hashimoto’s disease) and hyperthyroidism (Graves’ disease). Both of these thyroid diseases cause chronic inflammation, and the former one is a known factor associated with an increased risk of thyroid cancers. A rare case of a thyroid collision tumor was detected by Zhang et al. in a 64-year-old patient. This rare coexistence of primary thyroid mucosa-associated lymphoid tissue (MALT) lymphoma and papillary thyroid cancer was treated with a total thyroidectomy along with lymphadenectomy of the central compartment without any further signs of metastasis or tumor recurrence. Hashimoto’s thyroiditis is associated with both, MALT lymphoma and PTC. However, MALT lymphoma is less prevalent in the thyroid and more frequent in the stomach, often being caused by *Helicobacter pylori*-induced chronic inflammation.

This Research Topic describes well-known associations between the immune and endocrine systems in detail but also shows seemingly contradictory cases that uncover the hidden hormone regulation and signaling crosstalk. Understanding the interplay between these two systems is however necessary to propose new therapeutic strategies.
